# Estimation of Dietary Capsaicinoid Exposure in Korea and Assessment of Its Health Effects

**DOI:** 10.3390/nu13072461

**Published:** 2021-07-19

**Authors:** Youngjoo Kwon

**Affiliations:** Department of Food Science and Engineering, Ewha Womans University, Seoul 03760, Korea; Youngjoo.Kwon@ewha.ac.kr; Tel.: +82-2-3277-3103; Fax: +82-2-3277-4213

**Keywords:** capsaicin, capsaicinoids, chili peppers, consumption, dietary exposure, health effect, body weight, gastric distress

## Abstract

The consumption of capsaicinoids, the active components in chili peppers, has been associated with both positive and negative health effects, and the level of capsaicinoid exposure may be an important determinant. Dietary capsaicinoid exposure was estimated using a previously developed database for capsaicinoid content and a 24-h dietary recall dataset obtained from the Korea National Health and Nutrition Examination Survey. The estimated consumption level was evaluated to determine its potential effects on weight reduction and gastrointestinal distress. The estimated daily mean capsaicinoid intake was 3.25 mg (2.17 mg capsaicin), and most Koreans consumed 1–30 mg of capsaicinoids (0.67–20 mg capsaicin) in a day. No adverse effect of capsaicin consumption was reported other than abdominal pain. For long-term repeated consumption, 30 mg may be the maximum tolerable dose. However, the effects on body weight or energy balance were inconsistent in 4–12 week clinical studies conducted with various capsaicin doses (2–135 mg), which was likely due to the complex interplay between capsaicin dose, study length, and participant characteristics. Therefore, the capsaicin consumption of most Koreans was below the levels that may cause adverse effects. However, more long-term studies for the dose range of 2–20 mg are required to further characterize capsaicin’s health benefits in Koreans.

## 1. Introduction

Chili peppers (*Capsicum annuum* L.) are among the most popular spices worldwide. The spiciness of chili peppers stems from capsaicinoids, and the capsaicinoid content in chili peppers is directly related to its pungency [1]. The major capsaicinoids are capsaicin (8-methyl-*N*-vanillyl-6-nonenamide) and dihydrocapsaicin (8-methyl-*N*-vanillylnonanamide), which account for approximately 90% of all capsaicinoids [2].

In addition to their dietary intake, chili peppers have been applied topically to effectively manage pain. Capsaicin stimulates transient receptor potential vanilloid 1 (TRPV1), a nonselective cation that is activated by a wide variety of physical and chemical stimuli [3]. In contrast, exposure to high or repeated doses of capsaicin inhibits TRPV1, causing desensitization to its stimulants [4,5]. TRPV1 is expressed in a wide range of tissues, including skin, airways, and the gastrointestinal tract, as well as in different cell types such as urinary epithelial cells, pancreatic cells, and immune cells, thus highlighting the various biological roles of TRPV1 stimulants [6].

Chili pepper consumption has been suggested to provide various health benefits, including cancer-preventive, anti-inflammatory, and anti-obesity effects via TRPV1-dependent and TRPV1-independent mechanisms [6,7,8,9]. Clinical studies on these beneficial effects of chili pepper have mostly focused on the effects of its consumption on energy balance [10,11,12,13]. These studies have suggested that capsaicin intake may reduce obesity by decreasing food intake, increasing energy expenditure, and/or increasing lipid oxidation [12]. Dihydrocapsaicin, another major capsaicinoid in chili peppers, has been also shown to activate TRPV1 [14] and to have similar pharmacological effects as capsaicin [15,16]. However, most clinical studies have focused on capsaicin as an active compound of chili pepper and have documented its effects alone rather than in combination with other capsaicinoids such as dihydrocapsaicin.

TRPV1 is thought to be involved in both the control of food intake and energy expenditure [17]. TRPV1 can alter appetite and food intake by modulating appetite hormone levels or gastrointestinal vagal afferent signaling, which are important for determining meal size and duration [8,17]. Dietary capsaicin has been reported to increase thermogenesis via the stimulation of TRPV1 activation-mediated postprandial sympathetic nervous system (SNS) activity and increased catecholamine secretion in both humans and animals [18,19]. In response to TRPV1 excitation in afferent nerves (sensory neurons) upon exposure to capsaicin, efferent nerves (motor neurons) are excited to increase the secretion of catecholamine (e.g., epinephrine, norepinephrine, and dopamine) from the adrenal medulla [20]. Catecholamines secreted into the blood react with β-adrenergic receptors in peripheral organs, which enhance energy metabolism and consequently increase thermogenesis [21].

Nevertheless, capsaicin is also an irritant that can induce a burning sensation upon contact with any mucosa. Considering its irritant activity, there have been some concerns regarding the toxicity of capsaicin. Capsaicin can induce gastric distress such as acid reflux, and excessive chili consumption has been thought to be linked to gastric ulcers [22]. Consistent with this notion, a capsaicin-induced increase in satiety was attributed to gastrointestinal stress (high pain scores, burning sensation, nausea, and bloating) rather than modulation of the plasma concentrations of satiety-related hormones (e.g., glucagon-like peptide-1 and peptide YY) [23]. Moreover, a case-control study in Mexico City reported a significant increase in gastric cancer risk as the self-rated level of chili pepper consumption increased [24]. The cancer-promoting effect of capsaicin has also been reported in preclinical models of two-stage chemically induced skin cancer [25]. However, capsaicin is thought to inhibit rather than stimulate acid secretion by stimulating afferent neurons in the stomach and inhibiting the growth of *Helicobacter pylori* (*H. pylori*) [22]. A meta-analysis also indicated that dietary capsaicin can promote or prevent cancer depending on the dose [26]. Therefore, the beneficial effects or toxicity of capsaicin may depend on its consumption level.

Capsaicin intake has been estimated based on the overall consumption of major chili products (i.e., yields and export volumes) or the frequency of chili-pepper-containing product intake [27]. However, the association between capsaicin consumption and its health effects would be facilitated by more accurate estimations of capsaicin consumption [27]. Therefore, this study sought to estimate the dietary capsaicinoid (or capsaicin) exposure level in the Korean population and assess whether these consumption levels are linked to weight reduction and gastrointestinal distress. This study used the Capsaicinoid Content for Foods Commonly Consumed in Korea (CAPKO) database and the 24 h-dietary recall datasets obtained from the Korea National Health and Nutrition Examination Survey (KNHANES) to estimate the capsaicinoid consumption level in Korea. The CAPKO database includes data on the capsaicinoid content of various food products including chili peppers, red pepper powder, hot sauce, kimchi, red pepper paste, salted fish, instant noodles, and instant foods other than instant noodles. Additionally, the capsaicinoid level in red pepper powder, a major condiment of spicy food and a primary source of capsaicinoids in the Korean diet, was categorized into five levels to reflect the differences in their pungency depending on the composition of the pepper cultivar [27]. Afterward, the estimated capsaicinoid intake level was evaluated to determine its potential effects on weight reduction and gastric distress compared with previous results from the literature. Concretely, a literature search was conducted to identify clinical studies that examined the effect of capsaicin, capsaicinoids, or chili on body weight, body composition, energy metabolism, obesity, gastric distress, or gastrointestinal cancers. Crossover, randomized controlled, or case-control studies that examined energy expenditure, lipid oxidation, anthropometric measures, gastric symptoms, or gastric cancer as outcomes of interest were included. Studies were excluded if they were conducted only in children or included other interventions (e.g., exercise, other bioactive compounds) in addition to chili pepper or other capsaicin sources. Studies were also excluded if they did not provide data for capsaicin or capsaicinoid levels.

## 2. Materials and Methods

### 2.1. Study Design

This study was a cross-sectional analysis of the datasets obtained from the KNHANES in 2014–2018. This nationwide survey conducted by the Korea Center for Disease Control and Prevention collects information on the socioeconomic status, health-related behaviors, quality of life, anthropometric measures, and biochemical and clinical profiles of participants every year to assess the health and nutritional status of Koreans [28]. Each year’s survey included approximately 10,000 individuals aged one and older based on multi-stage clustered sampling to represent the entire Korean population. During the survey, food intake information was obtained using a 24-h recall (24 HR) interview [28].

### 2.2. Estimation of Capsaicinoid Intake

The combined 24 HR KNHANES datasets from 2014 to 2018 were examined to identify capsaicinoid-containing foods in the Korean diet. The capsaicinoid consumption level was calculated as previously described [29] based on food consumption data in the 24HR datasets and the CAPKO database [27]. Capsaicin and dihydrocapsaicin are the major capsaicinoids in chili peppers [1,2]. Although dihydrocapsaicin may have similar biological effects as capsaicin [15,16], most clinical studies have focused on capsaicin as the active compound in chili peppers. Therefore, existing datasets typically provide only the capsaicin dose rather than that of capsaicinoids. Capsaicin level was calculated based on the approximate proportion of capsaicin (two-thirds of capsaicinoids) for capsaicinoid level estimated in this study, to compare the capsaicin consumption levels reported in the literature [27].

### 2.3. Statistical Analyses

Data preparation and statistical analyses were conducted using the SAS software (version 9.4, SAS Institute Inc., Cary, NC, USA). The KNHANES datasets obtained from 2014 to 2018 were consolidated prior to analysis. A multi-stage sampling design was implemented, which consisted of combining the datasets, estimating the consumption level, and evaluating the statistical significance. Statistical analyses were performed after dividing the participants based on their capsaicinoid consumption level into very low capsaicinoid (VLC), low capsaicinoid (LC), moderate capsaicinoid (MC), high capsaicinoid (HC), and very high capsaicinoid (VHC) intake subgroups. Chi-squared tests were performed to evaluate the proportions of these capsaicinoid-intake subgroups in the study cohort and to compare the proportions of the consumed capsaicinoid sources (e.g., chili peppers, red pepper powder, red pepper paste) in the five capsaicinoid intake subgroups within each age and sex group. Multiple comparisons with Bonferroni correction were made to examine the differences in total energy intake and body mass index (BMI) among the capsaicinoid intake subgroups in each age and sex group. Multiple comparisons for total energy intake and BMI were also conducted with age adjustment for age groups lower than 20 years. Student’s *t*-test was performed to determine the differences in fat and sugar intake between the VHC and other capsaicinoid intake subgroups in participants aged 20 and older. *p*-values below 0.05 were considered significant.

## 3. Results

### 3.1. Estimation of Capsaicinoid Intake in the Korean Diet 

Red pepper powder is processed by drying and pulverizing Korean red chili peppers. This is a major condiment in Korean cuisine that is also largely used for the preparation of other capsaicinoid-containing foods such as kimchi, red pepper paste, and salted fish. The mean consumption of red pepper powder and fresh chili pepper was 4.93 and 3.55 g/day, respectively. Together, these two capsaicinoid sources account for approximately 5.5 g/day on a dry weight basis (data not shown). The capsaicinoid content in red pepper powder can vary depending on the red chili pepper varieties used to process the powder. To reflect the different pungency levels of commercially available red pepper powders, capsaicinoid content was estimated over five levels [27]. Red pepper powder with medium hotness is commonly consumed. However, extremely hot powder is also frequently purchased by consumers. Therefore, the capsaicinoid contents of red pepper powders with medium-hot and extremely hot intensity were used to estimate the mean intake and the maximum intake levels, respectively. When the capsaicinoid content of red pepper powder with medium-hot intensity (59.1 mg/100 g) was used to estimate capsaicinoid intake, the mean intake level was 3.25 mg/day and the mean intake per body weight was 0.052 mg/kg body weight (bw)/day (Table 1). In contrast, when the estimations were based on the capsaicinoid content of extremely hot red pepper powder (153.8 mg/100 g), the maximum daily intake was 118.01 mg and the maximum intake per body weight was 1.933 mg/kg bw/day (Table 1).

The average capsaicinoid consumption level was higher in males (3.94 mg/day) than females (2.49 mg/day), with female consumption levels being 63% of that in males (Table 1). However, this difference decreased when body weight was accounted for, with females (0.046 mg/kg bw/day) reaching 79% of the consumption level of males (0.058 mg/kg bw/day). In both males and females, mean intake per body weight was higher among those in their 40s (males: 0.065 mg/kg bw/day; females: 0.054 mg/kg bw/day) and 50s (males: 0.069 mg/kg bw/day; females: 0.052 mg/kg bw/day). Individuals aged 0–9 years had the lowest consumption levels (males: 0.036 mg/kg bw/day; females: 0.033 mg/kg bw/day) (Table 1). The maximum capsaicinoid consumption amounts in each sex and age group ranged from 8 to 17 times the mean intake per body weight in the same sex and age group (Table 1).

### 3.2. Characteristics of the High Capsaicinoid Intake Subgroup

Capsaicinoid intake levels exhibited a right-skewed distribution (i.e., the values were clustered around the left tail of the distribution). Depending on the level of capsaicinoid intake, participants were divided into five groups (VLC, LC, MC, HC, and VHC). The 25th (1 mg), 50th (3 mg), and 75th (5 mg) percentiles and a start point (12 mg) of extreme values in a long tail on the right side of the distribution were used as cut points. The average capsaicinoid intakes in the VLC (>1 mg), LC (1–3 mg), MC (3–5 mg), HC (5–12 mg), and VHC (≥12 mg) intake subgroups were 0.47, 1.93, 3.87, 7.06, and 17.32 mg/day, respectively (Table 2). The majority of Koreans (78%) consumed more than 1 mg capsaicinoid/day in the survey. The HC and VHC intake subgroups more frequently included males than females. In terms of age groups, the majority of male and female children (>70%) aged less than 10 years old were grouped in the VLC intake subgroup (Table 2). Male participants accounted for 20–30% of the LC, MC, and HC intake subgroups, whereas this proportion decreased to 16.3% in the VLC group and only 3.0% in the VHC intake subgroup. Individuals in the 10–20 year and ≥70 year age groups were more frequently included in the LC intake subgroup (approximately 40%). Female participants were more frequently included in the VLC (28.6%) and LC (41.1%) intake subgroups than males, while the HC and VHC intake subgroups contained only 10.3% and 0.9% of the overall female population, respectively. Except for the youngest female age group, where a majority (74.2%) was grouped in the VLC intake subgroup, the proportions of the capsaicinoid intake subgroups remained similar among females regardless of age groups (Table 2).

Based on the proportion of the capsaicinoid intake subgroups, the probable intake level was estimated to be 1–5 mg of capsaicinoids (0.67–3.33 mg capsaicin) per day, which coincided with the consumption levels for the LC and MC intake subgroups (Table 3). Further, the upper level of capsaicinoid intake was estimated to be 12.15–29.16 mg of capsaicinoids (8.10–19.44 mg capsaicin), which coincided with the consumption level for the HC intake subgroup (Table 3). However, it was assumed that individuals in the HC subgroup favor spicy foods and therefore likely consume extremely hot red pepper powder rather than medium-hot powder. Thus, the values were derived by multiplying the upper and lower consumption levels for HC by the ratio of the mean intake (7.91 mg) estimated with the capsaicinoid content of extremely hot red pepper powder to the mean intake (3.25 mg) estimated with the capsaicinoid content of medium-hot red pepper powder (Table 1).

**Figure 1:**
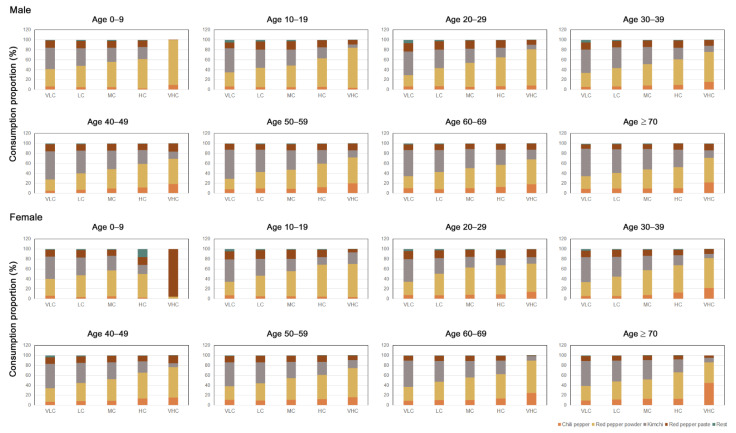
Consumption proportion of various capsaicinoid sources in the Korean diet in the five capsaicinoid intake subgroups in each age and sex group. The study participants were divided into five subgroups depending on the level of capsaicinoid intake: very low capsaicinoid (VLC), low capsaicinoid (LC), moderate capsaicinoid (MC), high capsaicinoid (HC), and very high capsaicinoid (VHC) intake.

The major sources of capsaicinoids were chili pepper, red pepper powder, kimchi, and red pepper paste (Figure 1). Other capsaicinoid sources included hot sauce, salted fish, instant noodles, and other convenience foods (e.g., spicy canned tuna and kimchi dumplings). However, the amount of capsaicinoid consumed via these food sources was very low, accounting for less than 1% of the total intake (Figure 1). From a population-wide perspective, red pepper powder was the most highly consumed (45%), followed by kimchi (30%), red pepper paste (13%), and chili peppers (10%) (data not shown). However, the consumption proportion of each capsaicinoid source varied depending on the capsaicinoid intake subgroup, age group, and sex. Except for females aged less than 10 in the VHC group, among whom red pepper paste accounted for approximately 96% of capsaicinoid consumption (Figure 1), red pepper powder was the main contributor to capsaicinoid consumption in the VHC intake subgroup. In males, the proportion of red pepper powder consumption was very high in children less than 10 years of age (90%) and 10–19 year-old participants (80%), as well as participants aged 20–29 years (72%). However, the percentage of red pepper powder consumption decreased (50–60%) among male participants aged 30 and older, and they exhibited higher red chili pepper consumption rates (15–20%) than the younger age groups (<10%). Among females of the VHC intake subgroup, red pepper powder (60%) and chili pepper (20%) were the major capsaicinoid sources. Moreover, individuals aged 70 and older consumed a higher proportion of chili peppers (45%) than red pepper powder (41%). In contrast, in the VLC intake subgroup, kimchi was the most consumed capsaicinoid-containing food (approximately 50%) regardless of age group in both males and females (Figure 1).

Multiple comparisons were made to examine differences in total energy intake and BMI among different capsaicinoid intake subgroups. Overall, the energy intake in the MC, HC, and VHC subgroups tended to be higher than those in the VLC and LC intake subgroups (Table 4). Therefore, the high capsaicinoid intake in the VHC group could have simply been a reflection of the higher food consumption rates of this subgroup. Total energy intake was significantly higher in the VHC subgroup than in the other subgroups among males in their 20s, 30s, 40s, 50s, and 60s (Table 4). Among females of the HC and VHC intake subgroups, only participants in their 40s exhibited a significantly higher total energy intake than the other subgroups. Given that age affects the energy requirements in the younger groups (<20 years), multiple comparisons were also performed with age adjustment. The significant differences remained the same after age adjustment in the younger age groups (Table 4). In contrast, BMI was not significantly higher in the VHC intake subgroups compared with the MC intake subgroups in males, and this was more apparent after age adjustment in the younger age groups, showing no significant differences among the capsaicinoid intake subgroups (Table 4). This finding was also observed in female participants except those in their 40s, whose VHC intake subgroup had significantly higher BMI than the VLC and LC intake subgroups.

Red pepper powder is often used alongside sugar and fat in food preparation. Student’s *t*-test was conducted to determine whether the sugar or fat intake in the VHC intake subgroup differed from that in the other intake subgroups in individuals aged 20 years and over. Fat intake was significantly higher in the VHC groups compared with all other capsaicinoid intake subgroups in males in their 20s, 30s, 40s, and 50s and females in their 20s and 40s (Table 5). Additionally, the VHC intake subgroup showed significantly higher fat intake than the other subgroups, except for HC, in males in their 60s and females in their 30s. Sugar intake was significantly higher in the VHC groups than in any of the VLC, LC, and MC subgroups in males. In contrast, sugar intake in females was significantly higher in the VHC intake subgroups than in the MC, LC, and VLC intake subgroups only in participants in their 40s and participants aged 70 years and over (Table 5).

## 4. Discussion

Capsaicinoid consumption has been linked to contradicting health effects such as cancer prevention, cancer promotion, weight reduction and gastric distress. Therefore, the health effects of dietary capsaicin might be largely determined by the exposure levels. Chili peppers are an essential ingredient in the Korean diet, which can lead to relatively high capsaicinoid consumption in the Korean population. This study utilized a previously developed database of capsaicinoid contents in foods commonly consumed in Korea (CAPKO) and the 24HR datasets obtained from the KNHANES, a nationwide survey program, to assess the dietary capsaicinoid exposure. Afterward, capsaicinoid intake levels were evaluated to determine its potential effects on weight control or gastric distress.

The average daily chili pepper consumption (combined consumption level of fresh chili pepper and red pepper powder) among the Korean population was estimated to be approximately 5.5 g (dry weight basis) in this study. In comparison, in an estimation based on production reports, chili pepper consumption per capita was estimated to be approximately 3 kg in South Korea in 2019, which is equivalent to approximately 8 g per day [30]. This difference may be attributed to food waste losses or loss during food preparation, which were not considered in estimates based on food-production data. A study also reported that the mean intake of red peppers was 4.6 g/day when three 24HR were conducted in 100 female university students [31], which was similar to the chili pepper consumption level estimated in this study; females in their 20s consumed approximately 4.2 g of chili peppers per day (77% capsaicinoid consumption relative to mean population capsaicinoid consumption) (Table 1).

The mean capsaicinoid intake was estimated to be 3.25 mg/day (2.17 mg capsaicin/day) (Table 1). Urinary metabolites of capsaicin were previously identified using HPLC in an attempt to utilize them as biomarkers to estimate capsaicin exposure [32]. However, there were individual variations in urinary metabolite levels, which were likely due to differences in capsaicin absorption and biotransformation [32], making it difficult to estimate dietary capsaicin levels based on urinary metabolite analysis. This study is the first estimation of capsaicinoid intake level in the Korean population. The mean capsaicinoid intake can be achieved by consuming approximately 300 g (three servings) of kimchi (a staple in Korean cuisine) in a day. This amount is also equivalent to 70 g of fresh Korean chili peppers (three fresh peppers) or 50 g (three tablespoons) of red pepper paste [27]. The majority of Koreans consume more than 1 mg of capsaicinoids per day (Table 2). The probable capsaicinoid consumption level was estimated to be 1–5 mg/day (0.67–3.33 mg capsaicin/day), whereas the upper consumption level was estimated to be 12–29 mg capsaicinoid/day (8.10–19.44 mg capsaicin/day) (Table 3). Therefore, Koreans consume more capsaicin than people in the US and Europe, where the maximum capsaicin intake was estimated to be approximately 1.5 mg/day, but less than the levels consumed in Thailand and Mexico, where capsaicin consumption was estimated to be 25–200 mg/day [33]. However, a small proportion of Koreans (<2%) consumed high levels of capsaicin (≥20 mg capsaicin) that were close to the levels in countries where chili pepper is extensively consumed.

Total energy intake was significantly higher in the higher capsaicin intake subgroups (HC and VHC) than in the lower intake subgroups (Table 4). In contrast, previous studies reported that capsaicin consumption reduced food intake [12,34,35]. SNS activation has been shown to be involved in the appetite-suppressing effect of capsaicin, and repeated exposure to capsaicin may induce TRPV1 desensitization [17]. This may also explain the fact that the appetite-suppressing effects of capsaicin were less pronounced in individuals who regularly consumed capsaicin before the trial [36]. Therefore, habitual consumption of capsaicin may alter the effects of capsaicin on appetite and food intake. Most Koreans begin consuming capsaicin-containing foods early in their life (<10 years old) and occasionally consume these foods thereafter. Therefore, the effects of capsaicin on appetite are likely only moderate or negligible in the Korean population.

Additionally, the higher energy intake in the higher capsaicin-intake subgroups in this study cohort was likely due to high food consumption. Spicy foods are frequently prepared with sugar and fat in Korean cuisine. The inclusion of fat and sugar along with red pepper powder in food preparation may contribute to the increased total energy intake in higher capsaicin intake subgroups. Consistent with this assumption, fat and sugar intake levels were also significantly higher in the VHC groups that exhibited significantly higher total energy intake compared to the other capsaicin intake subgroups in the same age and sex groups (Table 4 and Table 5). Therefore, dietary factors other than capsaicin could have significantly contributed to the high energy intake in the higher capsaicin intake subgroups in this study. In cases where other dietary factors were eliminated, for example, through the supplementation of 4 mg capsaicinoid in capsules for 3 months, food intake was reduced [13]. In a large population study that included 434,556 adults in China, high consumption of spicy foods was significantly associated with increases in BMI, body fat percentage, and waist circumference in both men and women [37]. However, the study did not evaluate the levels of energy or food intake and other dietary or behavioral habits related to obesity. In the study cohort examined herein, BMI was not significantly different between the higher and lower capsaicinoid intake subgroups, except for females in their 40s, despite the high total energy intake in the higher capsaicinoid intake subgroups (Table 4). Nevertheless, this was a cross-sectional study, and the study results may have not reflected individual eating habits, although individuals in the HC and VHC intake subgroups possibly exhibited a greater propensity to seek spicy foods. These observations may have been due to several confounding factors. For instance, the food intake of the study participants might have increased but these changes were not yet reflected in their BMI values. Furthermore, capsaicin intake might have helped manage body weight and body composition, thereby making an increase in BMI less apparent relative to the high energy consumption, as discussed below.

Capsaicin consumption may increase lipid oxidation and energy expenditure, and this may be related to capsaicin-induced thermogenesis. In rats, intravenous capsaicin injection increased thermogenesis, which was accompanied by a dose-dependent increase in catecholamine secretion [20]. Additionally, both catecholamine concentration and energy expenditure increased in non-obese healthy young men 30 min after consuming a meal containing 10 g of red pepper [38]. Further, this capsaicin-induced catecholamine secretion was abolished by administering adrenergic blockers such as propranolol [38]. Thus, the findings of previous studies suggest that capsaicin can increase thermogenesis via stimulation of the SNS in both humans and rodents. In clinical studies, a relatively high dose of capsaicin (6–30 mg/day) for a short-term (<24 h) was linked to increased lipid oxidation [19,39] and energy expenditure [19] after capsaicin intake. However, a single administration of a low dose of capsaicin (1–5 mg) resulted in different outcomes. Concretely, some reported no effect [40,41] whereas two other studies reported increases in lipid oxidation and/or energy expenditure [21,36]. Studies assessing repeated administration over a long period also yielded different outcomes. For instance, dietary supplementation with 33 mg capsaicin/day for 4 weeks did not affect energy expenditure or lipid oxidation [42]. In another study, a high capsaicin dose (135 mg daily capsaicin) was administered for 3 months to assess whether the treatment could promote weight maintenance after a 4-week very-low-energy diet that resulted in 5–10% weight reduction. Interestingly, in this study, capsaicin supplementation increased lipid oxidation and energy expenditure [11].

The effects of capsaicin on thermogenesis may be altered by the test dose and test period, as well as the characteristics of the participant population, such as their repeated exposure to capsaicin-containing foods, their maximum tolerable dose of capsaicin, and BMI. For example, the thermogenic components of SNS activity and energy expenditure were only significantly increased in lean but not obese women after a capsaicin-containing diet; however, both groups showed similar SNS activity levels at rest [21]. Additionally, the effect of capsaicin on postprandial thermogenesis was higher in study participants that did not regularly consume spicy foods compared to habitual users [36]. Capsaicin pretreatment was shown to prevent capsaicin-induced catecholamine secretion in animal studies [5]. Therefore, capsaicin adaptability may be related to the decreased activation of the SNS in individuals who regularly consume spicy foods [38]. Considering the effects of capsaicin sensitization, capsaicin might only affect energy expenditure for a short period and may not have any observable effects when administered long-term, which may explain why capsaicin intake (33 mg/day) for 4 weeks did not alter either energy expenditure or lipid oxidation [10,42]. However, another long-term (12 weeks) study with a higher dose (135 mg/day) demonstrated that regular capsaicin intake in non-users was effective in decreasing lipid oxidation during their weight maintenance period after weight loss [11]. Therefore, it is currently unclear whether high doses or longer exposure can overcome capsaicin adaptation. Additionally, participants in these long-term studies were overweight or obese, and the effect of capsaicin on SNS activity may only be apparent in individuals who underwent weight loss [11]. A meta-analysis suggested that the effects of capsaicin on energy expenditure or lipid oxidation may be dose-dependent, increasing at high (135–150 mg/day) and intermediate (20–35 mg/day) doses but having no effects at low (<7 mg/day) doses [12]. However, the aforementioned study did not consider the differences in study lengths, study participants, and types of capsaicin provided (tablet vs. meal). Therefore, capsaicin-induced thermogenesis may be altered by the interplay of various factors, including capsaicin dose, length or frequency of use, and anthropometric traits of the individuals, making it difficult to predict its population-wide effects.

Body composition change and/or weight reduction are ultimately important parameters that define the beneficial effects of capsaicin on weight control. However, dietary supplementation with 33 mg capsaicin/day for 4 weeks did not affect anthropometric measurements in obese individuals [42]. Additionally, the administration of a higher dose (135 mg daily capsaicin) for a 3-month weight-maintenance period following weight reduction did not suppress weight regain, even though capsaicin supplementation increased lipid oxidation and energy expenditure [11]. Another study called into question the rigor with which the covariates were controlled. For example, 4 mg of capsaicinoids for 3 months has been shown to only significantly reduce the percent body fat change and fat mass after post-hoc analysis in which the baseline values of body fat percentage, fat mass, and protein, fat, and carbohydrate intake were considered as covariates due to the involvement of multiple factors in body composition changes [43]. These observations are highly relevant to the present study, as the majority of Koreans consume 1–5 mg of capsaicinoids per day, and therefore it is not uncommon for Koreans to consume 4 mg of capsaicinoids per day. Moreover, as demonstrated in the present study, individuals that consumed high amounts of capsaicinoids also tended to consume higher amounts of food (Table 4 and Table 5). In long-term studies, food consumption may be difficult to control over long periods although capsaicin ingestion could be controlled because it was administered as capsules [11,13,42]. Therefore, it is important to consider food intake as a covariate, particularly in long-term studies. The observed effect of capsaicinoids on body fat could be a combination of the long-term effects of capsaicin on food intake, energy expenditure, and lipid oxidation, even though the SNS-stimulating effects of capsaicin may be reduced by repeated capsaicin exposure. Additionally, consuming capsaicin-containing meals (33 mg capsaicin/day for 4 weeks) reduced the postprandial insulin level by potentially increasing insulin sensitivity [10]. Notably, the decrease in postprandial insulin level after a capsaicin diet was more apparent with increasing BMI (≥26.3). Consistent with these findings, chili consumption has also been shown to decrease postprandial insulin levels in obese individuals [44]. TRPV1 has been shown to mediate glucose-induced insulin secretion, and TRPV1−/− mice have been shown to be more insulin-resistant than wild-type mice [45]. Therefore, the activation of TRPV1 after capsaicin intake may increase insulin sensitivity. However, the potential effect of a 4-mg capsaicinoid consumption level on insulin sensitivity or energy balance remains unclear. Further, its effect on other parameters related to metabolic mechanisms such as thermogenesis and lipid oxidation was not reported, and therefore more studies are required to verify the effect of capsaicinoid consumption on fat loss. Notably, only the aforementioned study instructed the participants to consume capsaicinoids before lunch [13,43]. In contrast, capsaicin capsules were consumed with each meal in the other two long-term studies [11,42]. Therefore, future studies should also determine whether taking capsaicinoids prior to a meal affects its effect on body weight or body composition.

Capsaicin is an irritant that elicits pain or a burning sensation upon contact with mucosal tissues. Therefore, topical application, inhalation, and dietary exposure to capsaicin can cause toxic effects associated with pain induction. The most common toxic effect of capsaicin ingestion is gastric distress [46]. However, capsaicin has also been studied for its potential therapeutic applicability in relieving gastric reflux-related symptoms due to its hypersensitizing and pain-relieving effects [4,47]. Esophageal infusion with a tabasco sauce suspension (0.84 mg capsaicin) increased the sensitivity to distension-induced secondary peristalsis, although the effect of capsaicin was reduced upon repeated exposure in both patients with gastroesophageal reflux disease (GERD) [48] and healthy volunteers [49]. Another study indicated that chili intake (chili capsules containing 1.46 mg capsaicin) could decrease early satiety in non-erosive gastroesophageal reflux disease (NERD) patients, which can be related to an increase in postprandial gastric accommodation after a capsaicinoid-containing meal compared to a placebo [50]. However, the same study also reported that chili could induce abdominal burning symptoms in NERD patients but not in healthy volunteers [50]. Additionally, capsaicin (5 mg) did not affect either gastric emptying or dyspepsia, but it increased postprandial abdominal pain in patients with heartburn [47]. In healthy individuals, 3 g of cascabel chili (2.64 mg capsaicin) but not ancho chili (1.46 mg capsaicin) significantly increased the number of reflux episodes and the percentage of time in which the pH in the esophagus is below 4 [51]. Therefore, studies have indicated that even low doses of capsaicin (<5 mg) can cause acute abdominal pain in healthy individuals [51] and aggravate abdominal burning symptoms or reflux in GERD patients and individuals prone to heartburn [47,50]. However, these studies that investigated the acute effects of capsaicin were relatively small and employed various capsaicin treatment regimens, making it difficult to compare them. The German Federal Institute for Risk Assessment also reported that capsaicinoids had no serious harmful effects, apart from the intolerance caused by allergies in adults after oral acute intake of chili fruits and their conventional preparations [52]. The authors reported that a dose of 5 mg capsaicin/kg bw can be assumed to represent the traditionally accepted maximum pungency of a meal consumed by adults [52]. The European Commission concluded that a safe dietary capsaicinoid exposure level could not be established based on the available data [33].

Long-term adverse effects may be more relevant to regular dietary exposure, as is the case in Koreans. Unfortunately, not all of the clinical studies reported the adverse effects of capsaicin intake in detail. Capsaicinoid exposure levels as low as 4 mg (approximately 2.6 mg capsaicin) may cause gastric distress in some individuals [13]. In another study, the acceptable dose was determined through a palatability test. Study participants that did not regularly consume capsaicinoid-containing foods could not sustain regular consumption of a higher-than-acceptable but tolerable dose (33 mg) of capsaicin over a long period, even though the adverse effects (gastric motility) disappeared after the first week of intake [42]. Among the participants that received the highest dose in the intervention (45 mg in each meal), all participants in the capsaicin supplementation group reported stomach burning following capsule consumption. Thus, 24% of the participants reduced their dose by half [11]. Therefore, capsaicinoid consumption over 30 mg may induce significant abdominal pain for most individuals and may not be consumable over long periods.

Few studies have investigated the relationship between chili consumption and the incidence of gastrointestinal cancer. However, some case-control studies have indicated that high chili consumption may increase the risk of gastric cancer [24,53]. In this study, the risk of gastric cancer was higher among high-level consumers (90–250 mg capsaicin/day) than among low-level consumers (0–29.9 mg capsaicin/day), and the findings were independent of *H. pylori* infection, which is a major risk factor for gastric cancer [53]. However, other factors such as sodium intake can also be attributed to the incidence of gastric cancer [22,54,55,56]. Additionally, individuals with high capsaicin consumption levels may also consume high amounts of foods, which can cause adverse health effects. Therefore, the association between chili consumption and gastrointestinal cancers requires more careful evaluation. The upper level of capsaicin intake was 8.10–19.44 mg/day and the maximum capsaicin intake level was 78.67 mg/day in this cohort. Therefore, the capsaicin consumption level in Koreans was similar to the low-level consumption in a case-control study conducted in Mexico [53]. Thus, even if the findings of the Mexican study were deemed true, only a few Korean individuals who repeatedly consume at the maximum level may be considered to be at a high risk of developing gastric cancer.

In this study, food intake was assessed via a food recall method and therefore assessment can carry recall bias and may not reflect long-term intake. Another limitation was the use of a database that does not cover all capsaicinoid-containing food items. However, the present study is the first to comprehensively estimate capsaicinoid intake based on a large-scale nutrition survey. Mean daily capsaicinoid consumption was estimated at 3.25 mg, and the majority of Koreans consumed 1–30 mg capsaicinoids per day. This level is not likely to cause adverse effects, however, more studies are required to confirm the population-wide effects of capsaicinoids on weight control at the aforementioned dose range. Notably, repeated consumption of capsaicinoids at a daily dose level of 4 mg was shown to reduce body fat, which is feasible for the Korean diet from a long-term perspective. However, these findings must be further validated in a larger study, and the underlying mechanisms of capsaicinoid-induced metabolic regulation must be further elucidated. Further, the consumption of other dietary components such as fat and sugar must also be controlled to achieve a robust assessment of the beneficial effects of capsaicin in weight loss and management.

## Figures and Tables

**Table 1:** Mean capsaicinoid intake and mean intake per body weight in different age groups in males and females.

	Intake (mg/day) ^(1)^					Intake Per Body Weight (mg/kg/day) ^(1)^			
Sex	Age Group	Mean ± SE	Max			Mean ± SE	Max		
Male	0–9	0.83 ± 0.03	16.19	3.94 ± 0.05	3.25 ± 0.03 (7.91 ± 0.07, 118.01) ^(2)^	0.036 ± 0.001	0.578	0.058 ± 0.001	0.052 ± 0.000 (0.127 ± 0.001, 1.933) ^(2)^
	10–19	2.95 ± 0.14	47.19			0.050 ± 0.002	0.682		
	20–29	3.75 ± 0.17	48.30			0.052 ± 0.003	0.759		
	30–39	4.55 ± 0.12	28.56			0.061 ± 0.002	0.473		
	40–49	4.80 ± 0.12	48.17			0.065 ± 0.002	0.641		
	50–59	4.73 ± 0.12	44.12			0.069 ± 0.002	0.748		
	60–69	4.02 ± 0.08	39.28			0.060 ± 0.001	0.600		
	≥70	3.19 ± 0.10	37.21			0.050 ± 0.002	0.770		
Female	0–9	0.74 ± 0.03	14.24	2.49 ± 0.03		0.033 ± 0.001	0.412	0.046 ± 0.000	
	10–19	1.91 ± 0.06	14.19			0.039 ± 0.001	0.378		
	20–29	2.50 ± 0.08	28.48			0.045 ± 0.001	0.483		
	30–39	2.73 ± 0.06	30.58			0.048 ± 0.001	0.499		
	40–49	3.09 ± 0.07	31.00			0.054 ± 0.001	0.454		
	50–59	2.95 ± 0.06	36.53			0.052 ± 0.001	0.473		
	60–69	2.71 ± 0.06	46.41			0.047 ± 0.001	0.802		
	≥70	2.06 ± 0.08	24.71			0.037 ± 0.002	0.472		

^(1)^ Estimates based on the capsaicinoid content of red pepper powder with medium-hot intensity (59.1 mg/100 g); ^(2)^ Estimates based on the capsaicinoid content of red pepper powder with extremely hot intensity (153.8 mg/100 g). Mean ± SE: mean intake, Max: maximum daily intake.

**Table 2:** Percentage (%) of participants in the five subgroups divided by their capsaicinoid consumption levels in different age groups in males and females.

Capsaicinoid Intake Subgroup ^(1)^	VLC	LC	MC	HC	VHC
Consumption level (mg/day)	<1	1–3	3–5	5–12	>12
Mean ± SE (mg/day)	0.47 ± 0.00	1.93 ± 0.01	3.87 ± 0.01	7.06 ± 0.03	17.32 ± 0.43
Weighted number of participants (percentage, %)	808,218,532 (22.1)	1,334,884,679 (36.5)	785,864,767 (21.5)	652,230,874 (17.8)	74,075,078 (2.0)
Male (%)	16.3	32.4	23.6	24.6	3.0
Age 0–9	72.3	22.8	4.1	0.7	0.1
Age 10–19	25.1	40.5	19.4	13.1	1.9
Age 20–29	16.7	35.7	21.5	23.6	2.5
Age 30–39	10.3	31.1	24.0	30.6	4.0
Age 40–49	7.0	28.4	28.9	31.3	4.4
Age 50–59	6.4	30.1	27.5	31.5	4.5
Age 60–69	9.6	33.9	27.9	26.9	1.7
Age ≥ 70	14.8	43.5	25.0	15.2	1.4
Female (%)	28.6	41.1	19.1	10.3	0.9
Age 0–9	74.2	22.9	2.5	0.3	0.0
Age 10–19	35.6	44.8	13.9	5.6	0.2
Age 20–29	29.4	39.5	20.3	9.9	0.8
Age 30–39	23.2	41.4	22.3	12.4	0.7
Age 40–49	16.8	43.7	23.0	14.9	1.6
Age 50–59	18.2	43.4	24.5	12.5	1.3
Age 60–69	22.9	43.4	21.3	11.7	0.8
Age ≥ 70	35.7	43.7	12.8	7.1	0.6

^(1)^ Study participants were divided into five subgroups depending on their level of capsaicinoid intake: very low capsaicinoid (VLC), low capsaicinoid (LC), moderate capsaicinoid (MC), high capsaicinoid (HC), and very high capsaicinoid (VHC) intake.

**Table 3:** Estimation of the probable capsaicinoid and capsaicin intake levels and upper intake levels.

	Probable Intake Level ^(1)^	Upper Intake Level ^(2)^	Maximum Intake ^(3)^
Capsaicinoid consumption level (mg/day/person)	1–5	12.15–29.16	118.01
Capsaicin consumption level ^(4)^ (mg/day/person)	0.67–3.33	8.10–19.44	78.67
Percentage (%)	58	18	

^(1)^ Consumption range for the low (LC) and moderate (MC) capsaicinoid intake subgroups. ^(2)^ The values were calculated by multiplying the consumption range for the high capsaicinoid (HC) intake subgroup with the ratio of the mean intake level (7.91 mg) estimated with the capsaicinoid content of extremely hot red pepper powder to the mean intake level (3.25 mg) estimated with the capsaicinoid content of medium-hot red pepper powder. ^(3)^ Maximum intake level when capsaicinoid intake level was estimated with the capsaicinoid content of extremely hot red pepper powder. ^(4)^ Capsaicin levels were estimated as two-thirds of the capsaicinoid levels.

**Table 4:** Total energy intake and BMI in the five capsaicinoid intake subgroups in each age and sex group.

	Capsaicinoid Intake Subgroup ^(1)^	VLC	LC	MC	HC	VHC
		**Total Energy Intake (kcal,** **Mean ± SE)**				
Male	Age 0–9	1482 ± 17.7 ^a(2)^	1910 ± 40.3 ^b^	2654 ± 167.7 ^c^	2800 ± 117.1 ^c^	1890 ± 205.4 ^ab^
	Age 10–19	2246 ± 42.7 ^a^	2367 ± 47.6 ^a^	3016 ± 142.2 ^b^	3318 ± 101.2 ^b^	4402 ± 474.1 ^b^
	Age 20–29	2120 ± 73.4 ^a^	2567 ± 66.1 ^b^	2785 ± 111.1 ^b^	3278 ± 105.7 ^c^	4192 ± 247.5 ^d^
	Age 30–39	2400 ± 96.3 ^a^	2408 ± 47.8 ^a^	2853 ± 80.1 ^b^	3193 ± 68.6 ^c^	4179 ± 235.2 ^d^
	Age 40–49	2267 ± 92.4 ^a^	2272 ± 40.4 ^a^	2597 ± 48.8 ^b^	2948 ± 55.4 ^c^	4297 ± 293.3 ^d^
	Age 50–59	2198 ± 85.6 ^ab^	2175 ± 43.9 ^a^	2428 ± 40.7 ^b^	2806 ± 46.7 ^c^	3381 ± 131.9 ^d^
	Age 60–69	1863 ± 49.5 ^a^	2050 ± 32.5 ^b^	2258 ± 40.1 ^c^	2631 ± 51.3 ^d^	3237 ± 219.5 ^d^
	Age ≥ 70	1685 ± 39.0 ^a^	1878 ± 30.6 ^b^	2112 ± 35.2 ^c^	2368 ± 70.9 ^d^	2740 ± 369.9 ^bcd^
Female	Age 0–9	1333 ± 15.7 ^a^	1713 ± 38.8 ^b^	1982 ± 87.0 ^c^	2196 ± 403.1 ^abc^	2060 ± 0.0 ^c^
	Age 10–19	1824 ± 36.2 ^a^	1950 ± 33.7 ^a^	2257 ± 78.6 ^b^	2658 ± 165.2 ^b^	3646 ± 565.8 ^b^
	Age 20–29	1688 ± 37.9 ^a^	1966 ± 53.9 ^b^	2399 ± 125.5 ^c^	2623 ± 130.4 ^c^	3819 ± 597.2 ^c^
	Age 30–39	1692 ± 32.8 ^a^	1854 ± 26.6 ^b^	2046 ± 36.9 ^c^	2236 ± 53.6 ^d^	2641 ± 222.7 ^cd^
	Age 40–49	1550 ± 38.9 ^a^	1749 ± 24.3 ^b^	1987 ± 40.5 ^c^	2252 ± 45.5 ^d^	2626 ± 172.5 ^d^
	Age 50–59	1581 ± 48.7 ^a^	1699 ± 20.3 ^a^	1929 ± 38.8 ^b^	2232 ± 55.9 ^c^	2364 ± 204.6 ^bc^
	Age 60–69	1495 ± 29.3 ^a^	1666 ± 21.9 ^b^	1945 ± 45.6 ^c^	2254 ± 67.3 ^d^	2097 ± 188.4 ^bcd^
	Age ≥ 70	1326 ± 24.4 ^a^	1493 ± 24.0 ^b^	1745 ± 41.6 ^c^	2047 ± 119.1 ^cd^	2467 ± 168.0 ^d^
		**BMI (kg/m^2^** **, Mean ± SE)**				
Male	Age 0–9	16.1 ± 0.07 ^a^*	16.9 ± 0.16 ^a^*	18.0 ± 0.53 ^a^*	18.8 ± 1.60 ^a^*	16.2 ± 0.58 ^a^*
	Age 10–19	20.9 ± 0.19 ^a^*	21.3 ± 0.19 ^a^*	21.2 ± 0.30 ^a^*	22.5 ± 0.31 ^a^*	22.1 ± 0.78 ^a^*
	Age 20–29	24.2 ± 0.26 ^a^	24.2 ± 0.27 ^a^	24.0 ± 0.28 ^a^	24.9 ± 0.37 ^a^	23.2 ± 0.78 ^a^
	Age 30–39	25.0 ± 0.24 ^a^	25.2 ± 0.20 ^a^	25.0 ± 0.20 ^a^	25.1 ± 0.18 ^a^	25.4 ± 0.52 ^a^
	Age 40–49	24.7 ± 0.31 ^a^	24.6 ± 0.15 ^a^	24.5 ± 0.17 ^a^	25.1 ± 0.17 ^a^	24.9 ± 0.41 ^a^
	Age 50–59	24.0 ± 0.28 ^a^	24.3 ± 0.13 ^a^	24.7 ± 0.17 ^a^	24.7 ± 0.15 ^a^	25.0 ± 0.37 ^a^
	Age 60–69	24.1 ± 0.22 ^a^	24.2 ± 0.15 ^a^	24.1 ± 0.14 ^a^	24.4 ± 0.15 ^a^	24.8 ± 0.45 ^a^
	Age ≥ 70	23.4 ± 0.20 ^a^	23.7 ± 0.14 ^a^	23.6 ± 0.21 ^a^	23.9 ± 0.23 ^a^	23.9 ± 0.46 ^a^
Female	Age 0–9	16.6 ± 0.15 ^a^	17.3 ± 0.24 ^a^	17.6 ± 0.45 ^a^	15.8 ± 0.57 ^a^	21.3 ± 0.00 ^a^
	Age 10–19	20.2 ± 0.16 ^a^*	20.3 ± 0.18 ^a^*	21.2 ± 0.44 ^a^*	21.0 ± 0.53 ^a^*	16.9 ± 0.55 ^a^*
	Age 20–29	21.6 ± 0.19 ^a^	21.8 ± 0.17 ^a^	21.6 ± 0.30 ^a^	21.7 ± 0.40 ^a^	21.1 ± 0.83 ^a^
	Age 30–39	22.4 ± 0.21 ^a^	22.5 ± 0.15 ^a^	22.6 ± 0.27 ^a^	22.5 ± 0.27 ^a^	24.6 ± 1.53 ^a^
	Age 40–49	23.0 ± 0.16 ^a^	23.0 ± 0.12 ^a^	23.3 ± 0.23 ^ab^	23.3 ± 0.23 ^ab^	25.4 ± 0.75 ^b^
	Age 50–59	23.6 ± 0.16 ^a^	23.6 ± 0.16 ^a^	23.8 ± 0.14 ^a^	23.8 ± 0.21 ^a^	24.0 ± 0.59 ^a^
	Age 60–69	24.3 ± 0.14 ^a^	24.2 ± 0.11 ^a^	24.1 ± 0.19 ^a^	25.0 ± 0.28 ^a^	24.1 ± 0.90 ^a^
	Age ≥ 70	24.4 ± 0.14 ^a^	24.6 ± 0.15 ^a^	24.7 ± 0.25 ^a^	25.0 ± 0.36 ^a^	24.2 ± 0.89 ^a^

^(1)^ Study participants were divided into five subgroups depending on their level of capsaicinoid intake: very low capsaicinoid (VLC), low capsaicinoid (LC), moderate capsaicinoid (MC), high capsaicinoid (HC), and very high capsaicinoid (VHC) intake. ^(2)^ Multiple comparisons with Bonferroni correction were made to examine the difference in total energy intake and body mass index (BMI) among capsaicinoid intake subgroups in each age and sex group. Analyses were also conducted with age adjustment for age groups under 20. Subgroups sharing the same letter superscript are not significantly different (*p* < 0.05). The asterisks indicate changes in significant difference after age adjustment.

**Table 5:** Fat and sugar intake levels in the five capsaicinoid intake subgroups in each age and sex group.

	Capsaicinoid Intake Subgroup ^(1)^	VLC	LC	MC	HC	VHC
		**Fat Intake (g, Mean ± SE)**				
Male	Age 20–29	59.4 ± 2.6 *^(2)^	68.9 ± 2.9 *	76.0 ± 4.5 *	92.4 ± 6.3 *	135.9 ± 14.3
	Age 30–39	66.1 ± 3.8 *	61.6 ± 1.9 *	71.7 ± 2.7 *	80.6 ± 3.1 *	111.4 ± 11.4
	Age 40–49	51.1 ± 2.4 *	52.1 ± 1.8 *	60.9 ± 2.0 *	66.4 ± 2.2 *	79.6 ± 5.7
	Age 50–59	48.2 ± 3.1 *	45.3 ± 1.9 *	48.6 ± 1.4 *	59.2 ± 1.9 *	81.8 ± 7.7
	Age 60–69	33.8 ± 2.6 *	36.2 ± 1.0 *	40.2 ± 1.4 *	46.7 ± 1.6	72.0 ± 14.7
	Age ≥ 70	26.9 ± 1.3	30.2 ± 1.0	34.2 ± 1.5	38.7 ± 2.3	62.7 ± 21.6
Female	Age 20–29	49.2 ± 1.8 *	55.7 ± 2.3 *	67.8 ± 5.0 *	72.4 ± 5.9 *	143.7 ± 31.5
	Age 30–39	45.5 ± 1.4 *	47.1 ± 1.1 *	50.4 ± 1.6 *	56.3 ± 1.8	85.4 ± 15.5
	Age 40–49	38.2 ± 1.5 *	41.0 ± 0.9 *	46.6 ± 1.6 *	58.2 ± 2.4 *	82.3 ± 11.3
	Age 50–59	36.0 ± 2.0	35.7 ± 0.8	38.1 ± 1.0	45.4 ± 1.6	49.7 ± 8.6
	Age 60–69	27.5 ± 0.9	30.4 ± 0.8	35.3 ± 1.2	44.0 ± 2.3	44.2 ± 9.1
	Age ≥ 70	19.6 ± 0.7	23.3 ± 1.0	28.4 ± 1.4	32.7 ± 3.1	35.1 ± 9.1
		**Sugar intake (g, Mean ± SE)**				
Male	Age 20–29	67.5 ± 5.4 *	72.8 ± 3.8 *	83.6 ± 8.6	88.5 ± 4.9	117.1 ± 17.6
	Age 30–39	81.5 ± 5.9	69.2 ± 3.0 *	72.6 ± 3.6 *	83.6 ± 3.9	114.6 ± 21.1
	Age 40–49	60.5 ± 3.9 *	64.0 ± 2.7 *	67.1 ± 2.9 *	71.7 ± 2.7 *	124.9 ± 15.0
	Age 50–59	70.1 ± 6.5	62.1 ± 2.5 *	65.7 ± 2.7 *	70.6 ± 2.6 *	88.3 ± 7.2
	Age 60–69	56.4 ± 3.9 *	58.0 ± 2.1 *	63.3 ± 2.4 *	76.1 ± 4.0	117.2 ± 26.5
	Age ≥ 70	44.6 ± 3.1 *	47.1 ± 2.1 *	63.3 ± 3.6	63.9 ± 4.1	79.8 ± 14.5
Female	Age 20–29	59.6 ± 3.1	61.3 ± 2.8	79.1 ± 5.9	80.2 ± 8.1	94.1 ± 17.6
	Age 30–39	57.1 ± 2.0	59.4 ± 2.0	61.5 ± 2.8	67.2 ± 3.4	73.1 ± 10.9
	Age 40–49	58.3 ± 3.0 *	57.6 ± 1.7 *	62.0 ± 2.4 *	72.5 ± 3.8	81.3 ± 6.3
	Age 50–59	60.5 ± 2.2	64.3 ± 2.0	69.1 ± 2.6	81.5 ± 5.1	57.0 ± 8.2
	Age 60–69	54.7 ± 2.4	56.3 ± 1.9	73.0 ± 5.7	70.5 ± 4.0	107.1 ± 44.8
	Age ≥ 70	45.6 ± 2.3 *	46.8 ± 2.3 *	49.0 ± 3.7 *	79.8 ± 15.1	76.6 ± 7.4

^(1)^ Study participants were divided into five subgroups depending on the level of capsaicinoid intake: very low capsaicinoid (VLC), low capsaicinoid (LC), moderate capsaicinoid (MC), high capsaicinoid (HC), and very high capsaicinoid (VHC) intake. ^(2)^ Student’s *t*-test was performed to determine the differences in fat and sugar intake between VHC and other capsaicinoid intake subgroups in male and female participants aged 20 or older. The asterisks indicate significant differences compared to the VHC intake subgroup (*p* < 0.05).

## Data Availability

Data are available from the official website of KDCA; https://knhanes.kdca.go.kr/knhanes/ (accessed on 18 July 2021).
